# Patients’ receptiveness for Medical students during consultation in Out patient department of a teaching hospital in Karachi Pakistan

**DOI:** 10.12669/pjms.292.3186

**Published:** 2013-04

**Authors:** Muhammad Laiq-uz-Zaman Khan, Masood Jawaid, Kamran Hafeez

**Affiliations:** 1 Muhammad Laiq-uz-Zaman Khan, FCPS, Dow University Hospital, Karachi, Pakistan.; 2 Masood Jawaid, FCPS, Dow University Hospital, Karachi, Pakistan.; 3 Kamran Hafeez, FCPS, Dow University Hospital, Karachi, Pakistan.

**Keywords:** Patients’ satisfaction, Patient based teaching, Ambulatory teaching

## Abstract

***Objective:*** Patients’ attitude towards medical students’ presence during treatment depends on the cultural values of the society. This study was conducted to find out the patients’ receptiveness in our society to be involved in teaching process for medical students during consultation in out patient department of a teaching hospital in Karachi Pakistan.

***Methodology:*** This cross sectional study was conducted in the surgical Out Patient Department (OPD) at Dow University Hospital from May 2012 to June 2012. Four hundred and eleven patients consented for participation through non probability purposive sampling, in which 279 patients were from morning clinics in the presence of students for clinical teaching, while 132 patients participated through evening clinics of surgery, when students were not present for comparison in specific dimensions of care for patients’ satisfaction.

***Results:*** Majority of patients 293 (71%) agreed with the teaching of students during consultation and they feel they are contributing in future doctor’s teaching, only 24% patients disagreed. Fifty two percent of patients who disagreed reported interference in privacy, 34% reported interference in consultation and 43% felt it resulted in prolong waiting time due to teaching.

***Conclusion:*** Majority of the patients agree to be part of teaching for medical students and this study can be used to assess the educational interventions designed to improve the patient based teaching.

## INTRODUCTION

Clinical medicine is a practical field related to human being and requires certain skills in psychomotor and affective domain along with cognitive development and to acquire these skills real patient encounter is important.^1^^-^^3^ Patient based teaching is an ethical issue and requires patient’s consent and cooperation.^4^^,^^5^

There is a pressure over health care providers and hospital managers in changing climate of health services to evaluate the services in view of patient’s expectations and their mismatch can result in patient’s dissatisfaction.^6^^,^^7^ These days medical education has been shifted more to the out patient settings (ambulatory teaching) from hospitals^8^ but there is a perception that due to concerns of patient’s discomfort; many hospitals are reluctant to participate in medical education of the students especially in outpatient settings. Presence of students during consultation in out patient clinics results in increasing waiting time for consultation and perception of interference in privacy and consultation process.^4^^,^^9^ To find out the patient’s attitude towards medical students involvement during treatment, many studies had been conducted abroad and results show that only minority of patients refuse student’s presence during treatment.^1^^,^^2^^,^^5^^,^^10^^,^^11^

Patients’ attitude towards medical students’ presence during treatment depends on the cultural values of the society^12^^,^^13^. As such studies done elsewhere cannot be as applicable as these are at the site where the study is conducted to improve patients’ satisfaction we need local studies to understand our society expectations for planning and evaluation of health services and medical education. There are studies^1^^,^^2^^,^^5^^,^^10^^,^^11^ conducted in different countries but to the best of authors’ knowledge, no locally conducted study was found on literature search to cover this aspect of patients’ satisfaction except one which was conducted generally on patients’ satisfaction with hospital services.^9^ This study was conducted on this aspect of patients’ satisfaction with consultation in the presence of medical students for teaching in our hospital, which provides health services to different socioeconomic groups of the society along with teaching of medical students.

## METHODOLOGY

This was a cross sectional study conducted in the surgical Out Patient Department (OPD) at Dow University Hospital from May 2012 to June 2012. Four hundred and eleven patients consented for study through non probability purposive sampling of the patients in which 279 patients participated in morning timings in the presence of students for clinical teaching, while 132 patients participated through evening clinics of surgery when students were not present for comparison of results in specific dimensions of care for patients’ satisfaction.

A self-administered structured questionnaire was developed in English. It consisted of 10 questions in two main sections. First section had a set of six questions about the socio-demographic characteristics which were gender, age, marital status, educational level, current occupation and residence. In the second section they were asked questions about their feelings regarding the practice of consultation in morning clinics in the presence of students and being involved in student teaching and their reasons for non-acceptability. Same questionnaire was used for patients’ satisfaction on same dimensions of care in evening clinics in the absence of students and teaching activity. They had the option of giving a free-text response or ticking one or more options. Their response for the question of students’ presence during consultation was captured using a Likert scale (1 = strongly disagree to 5=strongly agree), while responses for other dimensions of care during consultation were captured to evaluate the patients’ response regarding students’ presence in the background of patients’ over all satisfaction with consultation. The study was conducted at the end of fifth semester when students had already attended three months of clinical training in different departments during their clinical rotation.

Patients were unaware till they arrived at the clinic room that their consultation would include medical students teaching. To obtain informed consent for participation in the study and data collection through questionnaires after consultation a research assistant was present in the waiting area. Data was analyzed with statistical package for social sciences (SPSS) version 16. Descriptive statistics was used to present frequencies and percentages and statistical significant difference was noted with the application of chi X^2^ test with a two-tailed p-value <0.05.

## RESULTS

Four hundred and eleven patients in which one hundred thirty two were from evening clinics participated in this study with 12 teaching sessions in morning surgical OPD in the period of May to June 2012, nine patients who refused to participate didn’t include in the study and they refused because of the language problem, ill health or lack of time. Demographic data of the patients is shown in Table-I.

**Table-I:** Demographic Data of the patients. n= 411 (%)

Sex		
	Male	242 (59)
	Female	169 (41)
Age		
	>40 Years	214 (52)
	<40 Years	197 (48)
Residence		
	Urban	300 (73)
	Rural	111 (27)
Education		
	No education	82 (20)
	Primary	78 (19)
	Secondary	115 (28)
	Graduate	119 (29)
	Postgraduate	16 (04)

**Fig.1:**
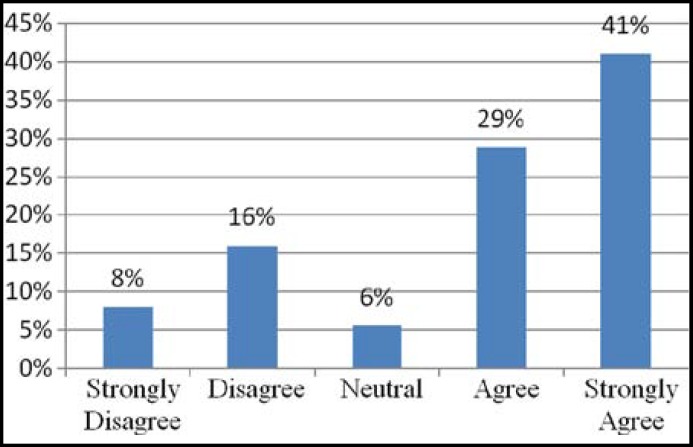
Patients’ receptiveness of their own involvement in student teaching

**Fig.2:**
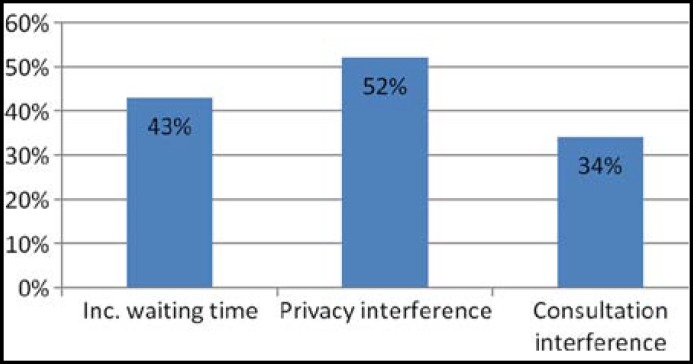
Patients perception for dissatisfaction in presence of students (n= 99 Patients

**Table-II:** Patients’ satisfaction for different dimensions of consultation in a teaching hospital OPD

*Dimension of care*	*Morning OPD* *(with students)* *N = 279 *		*Evening OPD* *(without students)* *N = 132 (%)*		*X* ^2^	*P*
	*Agree and Strongly agree* *n (%)*	*Neutral* *n (%)*	*Agree and Strongly agree* *n (%)*	*Neutral* *n (%)*		
Explanation of care	240 (86)	06 (02)	117 (89)	05 (04)	2.52	0.28
Comfort asking questions	226 (81)	07 (03)	116 (88)	07 (05)	8.82	0.01
Respect for privacy	212 (76)	14 (05)	111 (84)	12 (09)	11.91	0.002
Waiting for consultation	170 (61)	25 (09)	94 (71)	08 (06)	4.17	0.12
Agree with students’ presence	212 (76)	14 (05)	79 (60)	09 (07)	11.62	0.003
Overall consultation satisfaction	215 (77)	17 (06)	111 (84)	04 (03)	3.11	0.211

Four hundred and eleven patients, who consented, got completed questionnaires by research assistant after consultation while 9 patients refused to participate. Sixty seven (16%) had previous experience of medical students teaching during their consultation. In 60% of consultations students were involved for history taking and 73% of patients accepted students without any objection while in 47% of consultations students examined patients alone or in the presence of consultant with patients’ consent and acceptance rate was 61%. Majority of patients (71%) agree or strongly agree with the teaching of students during consultation and they feel they are contributing in future doctor’s teaching, only 24% patients disagreed as shown in Fig.1. Those patients who disagreed with presence of student during consultation (24%) and their perceived reasons of disagreement are shown in Fig.2. while 5% responded neutral on Likert scale for presence of medical students. Comparison of different dimensions of patients’ satisfaction during consultation in the presence of students in morning clinic and in their absence in evening clinics is shown in Table-II and these results show no statistically significant difference (P > 0.05) in patient satisfaction with the presence and absence of students during consultation.

## DISCUSSION

Our study shows that majority of patients 293 (71%) agree with the teaching of students during consultation and they feel they are contributing in future doctor’s teaching, only 24% patients disagreed because of their perception of interference in consultation process, privacy interference or prolong waiting time. Acceptability rate for physical examination was low up to 61% and for history taking it was 73%. Salisbury K. and associates reported acceptance of students in the presence of supervisor 70.4% for physical examination and 81.8% for history taking in Australian general practice setting.^11^ Hajioff D and Birchall M. reported in their study 09% non acceptance of medical students in ENT OPD along with 14% patients wanted to spend some time alone with doctor without students.^1^ One study conducted locally in a private teaching hospital by Qidwai and associates for patients’ satisfaction with hospital services reported 42% non acceptance for medical students in which 49% of them feel interference in privacy and 4.5% for consultation interference^9^, while in our study 52% of patients who disagreed reported interference in privacy, 34% interference in consultation and 43% prolong waiting time due to teaching. Patients in morning clinics have more acceptance ratio for students compared to evening clinics (P= 0.003) which shows that morning patients already expect presence of students before attending the clinic in a teaching hospital. Although 71% patients agree for students presence but when we see the patients satisfaction ratio for over all consultation, there is no statistically significant difference in presence and absence of students (P= 0.21).

In the real world of clinical practice medical students need to have experience of real patients to develop clinical skills and the respective community view regarding their involvement of their consultation as teaching tool for medical students according to their cultural values.^12^^,^^13^ Patient based teaching is an ethical issue and requires patient’s consent and cooperation.^4^^,^^5^ It is of particular interest for medical educators that how to involve patients in teaching process.^14^ Studies done in different countries show generally patients enjoy involvement in teaching.^1^^,^^2^^,^^5^^,^^10^^,^^11^ It is also important to realize that even though majority of patients have positive feelings for teaching over them but when a group of students visit, occasionally results in feelings of being on display, uncomfortable and nervous as reported by a previous study that 8% of patients felt that only fewer physicians should be at the time of physical examination.^15^

This study provide a stage for further research in our cultural setup, it is not known how far our results can be generalized to other specialty clinics like gynecology, psychiatry, which require special consideration. Although in our setup no significant number of studies are available but on the basis of patients’ response in this survey we can make some suggestions. We agree with the recommendation of Hajioff D and Birchall M.^1^ that those patients who are not willing for consultation in the presence of students have a significant minority (24%) and they have a right to see the doctor alone. Up to four students are acceptable to patients, although other studies suggested a limit of two to three.^16^ Students should be active participants in clinics rather than passive observers that may require restructuring of clinical teaching. To increase the role of out patient clinics in medical education further research over these issues is required.

## CONCLUSION

In conclusion our study expands on earlier studies conducted abroad to develop suggestions for involvement of patients in teaching process and to develop more patient based teaching we must incorporate patients’ insight in our practice of clinical teaching. In the process of teaching active engagement of patients for sharing of their experiences of illness may be more rewarding for them and patients have much to teach us about their illness. Using simulation and standardization should also be considered to address those scenarios in which patients are unwilling to allow students to participate. Majority of the patients agree to be part of teaching for medical students and this survey can be used to assess the educational interventions designed to improve the patient based teaching.
